# Protective Effect of Nicotinamide Riboside on Glucocorticoid-Induced Glaucoma: Mitigating Mitochondrial Damage and Extracellular Matrix Deposition

**DOI:** 10.1167/iovs.65.8.1

**Published:** 2024-07-01

**Authors:** Nan Zhang, Pengyu Zhang, Xizhi Deng, Min Zhu, Yixin Hu, Dongxiao Ji, Lufan Li, Yang Liu, Wen Zeng, Min Ke

**Affiliations:** 1 Department of Ophthalmology, Zhongnan Hospital of Wuhan University, Wuhan, Hubei, China; 2 Department of Ophthalmology, Huaihai Hospital of Henan University, Kaifeng, Henan, China

**Keywords:** glucocorticoid-induced glaucoma (GIG), trabecular meshwork (TM), nicotinamide riboside (NR), extracellular matrix (ECM), mitochondria

## Abstract

**Purpose:**

Glucocorticoid-induced glaucoma (GIG) is a prevalent complication associated with glucocorticoids (GCs), resulting in irreversible blindness. GIG is characterized by the abnormal deposition of extracellular matrix (ECM) in the trabecular meshwork (TM), elevation of intraocular pressure (IOP), and loss of retinal ganglion cells (RGCs). The objective of this study is to investigate the effects of nicotinamide riboside (NR) on TM in GIG.

**Methods:**

Primary human TM cells (pHTMs) and C57BL/6J mice responsive to GCs were utilized to establish in vitro and in vivo GIG models, respectively. The study assessed the expression of ECM-related proteins in TM and the functions of pHTMs to reflect the effects of NR. Mitochondrial morphology and function were also examined in the GIG cell model. GIG progression was monitored through IOP, RGCs, and mitochondrial morphology. Intracellular nicotinamide adenine dinucleotide (NAD^+^) levels of pHTMs were enzymatically assayed.

**Results:**

NR significantly prevented the expression of ECM-related proteins and alleviated dysfunction in pHTMs after dexamethasone treatment. Importantly, NR protected damaged ATP synthesis, preventing overexpression of mitochondrial reactive oxygen species (ROS), and also protect against decreased mitochondrial membrane potential induced by GCs in vitro. In the GIG mouse model, NR partially prevented the elevation of IOP and the loss of RGCs. Furthermore, NR effectively suppressed the excessive expression of ECM-associated proteins and mitigated mitochondrial damage in vivo.

**Conclusions:**

Based on the results, NR effectively enhances intracellular levels of NAD^+^, thereby mitigating abnormal ECM deposition and TM dysfunction in GIG by attenuating mitochondrial damage induced by GCs. Thus, NR has promising potential as a therapeutic candidate for GIG treatment.

For decades, glucocorticoids (GCs) have been used as potent anti-inflammatory and immunosuppressive medications in clinical settings.^1^ However, prolonged use of GCs poses the risk of developing side effects. Glucocorticoid-induced glaucoma (GIG) is a common complication, resulting in irreversible vision loss and impeding the widespread and safe utilization of GCs.^2^ The trabecular meshwork (TM) functions as the primary pathway for aqueous humor drainage.^3^ The prevailing belief is that the extracellular matrix (ECM) in the TM juxtacanalicular region contributes predominantly to the outflow resistance in the anterior segment.^3^ GCs have the potential to induce excessive ECM deposition and TM dysfunction, thereby elevating the resistance of the TM outflow pathway. This, in turn, leads to consistently elevated intraocular pressure (IOP) and the death of retinal ganglion cells (RGCs).^4^ Beyond GIG, the pathogenesis of primary open-angle glaucoma also involves the obstruction of aqueous humor outflow due to abnormal TM stiffness and function.^5^ Therefore, understanding the pathological alterations in TM is crucial for the development of effective therapeutic interventions.

Accumulating evidence points to the essential role of mitochondria in the pathogenesis of GIG.^6^^,^^7^ Our team has additionally identified abnormal mitochondrial fragmentation in TM as the main pathological change associated with GIG.^6^ Prolonged exposure to GCs induces mitochondrial damage, encompassing mitochondrial dysfunction, abnormal mitochondrial structure, and increased reactive oxygen species (ROS) generation.^8^ Furthermore, research has confirmed the presence of the GC receptor (GR) in mitochondria, validating the direct effect of GCs on mitochondria.^9^

Accumulating evidence indicates a close interconnection between mitochondria and the ECM across various tissues and organs.^10^ Mitochondrial respiratory chain dysfunction is a pivotal factor in promoting ECM integrity.^11^ Furthermore, mitochondrial dysfunction and metabolic reprogramming may lead to reduced resilience and heightened susceptibility to profibrotic responses in idiopathic pulmonary fibrosis.^12^ Evidence suggests that impaired mitochondria induce inflammatory processes and fibrosis progression, thereby enhancing ECM production.^13^ Previous research from our study reported that rapamycin treatment could attenuated autophagy dysfunction, also prevented mitochondrial damage and conferred protection to TM in GIG.^14^ However, it did not delve into the detailed effect of dexamethasone (DEX) on the mitochondria of the TM.^14^ Therefore, this study hypothesized that alleviating mitochondrial damage could potentially prevent GIG progression.

Nicotinamide riboside (NR) is a precursor of nicotinamide adenine dinucleotide (NAD^+^), an essential metabolite involved in numerous biological processes.^15^ Crucially, NAD^+^ plays a vital role in the mitochondrial respiratory chain,^16^ and NAD^+^ levels also contribute significantly to ECM organization.^17^ NR, a form of vitamin B3 present in the diet and bioavailable to humans,^15^ is well tolerated in long-term oral supplementation and effectively stimulates NAD^+^ metabolism in healthy adults.^18^ NR enhances mitochondrial function and improves mitochondrial respiration.^19^^–^^21^ Moreover, NR exhibits a noteworthy therapeutic effect in fibrotic diseases.^21^^–^^23^ Our previous reports highlighted the protective effects of NR on RGCs in various glaucoma models, including acute RGC injury (optic nerve crush), chronic ocular hypertension, and inherited glaucoma mouse models.^24^^–^^26^ Nicotinamide, a precursor of NAD^+^, has been documented to enhance inner retinal function and promote ocular blood supply in individuals with glaucoma, indicating the potential neuroprotective utility of NAD^+^ in the management of this condition.^27^^,^^28^ Furthermore, a clinical trial is currently being conducted to examine the potential protective effects of NR in individuals diagnosed with primary open angle glaucoma. The study posits that NR treatment may attenuate the progression of retinal nerve fiber layer thinning and visual field deterioration when compared to a placebo group over a 24-month period of observation.^29^

A previous study has demonstrated a dose- and time-dependent reduction in the NADPH signal caused by H_2_O_2_ in cultured human TM cells.^30^ Notably, cells with higher initial NADPH fluorescence exhibited greater resilience to peroxide-induced viability loss. This study suggested that intracellular NADPH may play a crucial role in the response to oxidative insult in HTM cells and contribute to the age-dependent risk of developing primary open angle glaucoma, indicating that NAD^+^ precursors’ administration might and could elevate the intracellular NAD^+^/NADP^+^ level and increase the tolerance of TM to oxidative stress.^30^ However, the role of NAD^+^ precursors in the TM has not yet been explored. Collectively, these findings suggest that NR could be a promising candidate for glaucoma treatment. However, the specific role of NR in TM remains unknown for GIG.

Given the substantial protective effect of NR on mitochondrial damage and fibrosis-related disorders, this study hypothesized that NR has the potential to mitigate impaired ATP synthesis, decrease in mitochondrial membrane potential, and ECM accumulation in the TM of GIG. To test this hypothesis, this study conducted investigations into the protective effects of NR on TM in both ex vivo and in vivo GIG models. This study aimed to elucidate the role of NR protection in the TM of GIG.

## Methods

### Animals

Adult C57BL/6J mice (both male and female mice, aged = 6–8 weeks, weight = 18–25 g) were purchased from the Animal Center of Wuhan University. Animal procedures were in strict agreement with the guidelines of the ARVO Statement for the Use of Animals in Ophthalmic and Visual Research and were approved by the institutional animal care. All animals were housed in a specific environment maintained at 20°C to 25°C with a 12-hour light/12-hour dark cycle, with free access to food and water. Mice were randomly divided into different treatment groups. The GIG mouse model was built as previously described.^6^^,^^14^^,^^31^ Briefly, dexamethasone (acetate; DEX-Ace, A45645; OKA, Shanghai, China) was injected as a subconjunctival administration into the right eye of each mouse (10 mg/mL, 10 µL/eye, once weekly). Only mice with IOP elevated by ≥4 mm Hg above baseline were included in this study. NR (Niagen; ChromaDex, Inc., Longmont, CO, USA) treatment was administered as previously described (1000 mg/kg mouse weight, dissolved in sterilized phosphate-buffered saline [PBS], every 2 days, via intraperitoneal injection) and started at the same time as DEX-Ace.^24^^,^^26^ Mice in the control group received sterilized PBS.

### Primary Human Trabecular Meshwork Cells

Primary human TM cells (pHTMs) were isolated from the postmortem donor eyes in compliance with the Declaration of Helsinki, approved by the Medical Ethics Committee of Zhongnan Hospital, Wuhan University. The cell separation and identification protocol followed previous methodologies.^32^ The pHTMs were obtained from 5 donors aged 19, 31, 45, 47, and 77 years, all without a known history of ocular disease. Myocilin and matrix Gla protein (MGP) were used for cell identification (Supplementary Fig. S1).^33^^,^^34^ Cultivation of pHTMs occurred in DF12 medium (HyClone, USA) supplemented with 10% fetal bovine serum (Gibco, USA) and 1% penicillin/streptomycin (Beyotime, Biotechnology, China) at 37°C with 5% CO_2_, and the medium was refreshed every 24 hours. Passages 5 to 7 were used for the study.

For ex vivo experiments, DEX (D4902; Sigma-Aldrich, Darmstadt, Germany; 100 nM) was added to establish the GIG cell model (Supplementary Fig. S2). To determine the appropriate NR (HY-123033A; MedChemExpress, stock solution = 150 mM NR in DMSO, working solution = 10 mM in PBS) concentration, pHTMs were treated with different concentrations of NR, and cell viability was assessed using a CCK-8 assay at 24 and 72 hours after NR treatment (Supplementary Fig. S3). Specifically, NR (400 µM) was co-added to the pHTMs medium simultaneously with DEX.

### Western Blotting

Cultured pHTMs were lysed with radioimmunoprecipitation assay lysis buffer containing protease (1:100) and phosphatase inhibitor (1:100) cocktails for 30 minutes on ice.^14^ Briefly, equal amounts of protein from each group (10 µg) were separated by sodium dodecyl sulfate–polyacrylamide gel electrophoresis and electro-blotted onto FFP24 membranes (Beyotime Biotechnology, China). After 2 hours of incubation in 5% bovine serum albumin (BSA) at room temperature, the membranes were incubated with primary antibodies (1:1000 for all primary antibody; rabbit anti-fibronectin [FN], NBP191258, Novus Biologicals; rabbit anti-collagen Ⅳ [COL4]A1: A10710, ABclonal Technology; rabbit anti-Myocilin: A1589, ABclonal Technology; rabbit anti-Myocilin, abs115636, Absin, China) overnight at 4°C, followed by incubation with goat anti-rabbit IgG secondary antibody (1:5000; AS014; ABclonal, Wuhan, China) for an additional 2 hours. Membranes were developed with enhanced chemiluminescence using Tanon 5200 ECL (Tanon, China), and protein expression levels were semi-quantified using ImageJ software (version 1.8.0). GAPDH (mouse anti-GAPDH [1:5000; 60004-1-Ig, Proteintech]) with goat anti-mouse IgG secondary antibody (1:5000, AS003, ABclonal Technology, China) served as the loading control.

### Immunofluorescence

The pHTMs were fixed, permeabilized, and blocked for 2 hours in a blocking buffer containing 1% BSA. Subsequently, they were incubated with primary antibodies (1:200, FN, COL4) at 4°C overnight and secondary antibodies (1:200; goat anti-rabbit Alexa Fluor 488 [GB22403, Servicebio], and anti-rabbit Alexa Fluor 594 [GB21403, Servicebio]) for 2 hours at room temperature. Nuclei were stained with 4′,6-diamidino-2-phenylindole (DAPI; Sigma, D9542). Microscopy (Olympus, Japan) and ImageJ software were used for image acquisition and analysis, respectively. The average fluorescence intensity per cell was used for statistical analysis.

### pHTMs Function

#### Phagocytosis Assay

The pHTMs from each group were transferred to a serum-free medium and incubated with pretreated fluorescent beads (L2778; 1.0 µm; diluted 50,000 times; Sigma-Aldrich, Darmstadt, Germany) at 37°C for 2 hours. Flow cytometry was used for measurement, and FlowJo software applications were utilized for data analysis. The blank group (treated with serum-free medium only without microbeads) was set for distinguishing between cells that have phagocytosed microbeads and those that have not. ECD-A channel value of 10^4^ was established as the threshold.

#### Proliferation

Cell proliferation was assessed utilizing the BeyoClick EdU cell proliferation kit with Alexa Fluor 594 (Beyotime, Shanghai, China). The pHTMs from different groups were cultured for 3 days, then exposed to 10 µM 5-ethyl-2′-deoxyuridine (EdU), and incubated at 37°C for 6 hours. Fluorescence microscopy (IX71; Olympus, Tokyo, Japan) was used for cell visualization, and ImageJ software was used for analysis.

#### Transwell Assay

Cell migration ability was determined by Transwell assay. Cells were digested with 0.25% trypsin (Gibco, Thermo Fisher Scientific, Waltham, MA, USA) and resuspended in serum-free medium, then seeded in the upper chambers (Falcon, REF#353097, Corning, NY, USA) filled with a nonserum medium (200 µL of cell suspension). The lower chambers contained 500 µL complete medium with 15% serum. The setup was incubated at 37°C in 5% CO_2_ for 5 hours. Cells in the lower chamber were fixed with 4% PFA for 15 minutes at room temperature, stained with 0.1% crystal violet (Beyotime) for 30 minutes, and observed and analyzed, respectively, using an inverted microscope (IX71; Olympus, Tokyo, Japan) and ImageJ.

### Mitochondrial Morphological Analysis

Mitochondria of pHTMs were labeled with MitoTracker (Red) FM probe (M7514; Thermo Fisher Scientific, USA) following the manufacturer's instructions. Briefly, the cells were incubated with the MitoTracker probe at a final concentration of 200 nM at 37°C for 150 minutes. Mitochondrial images were captured using a Leica TCS SP8 X (Leica, Germany) under 63 × magnification. ImageJ was used for the quantification of mitochondrial branch length following previous methodologies reported by other researchers.^35^

### Mitochondrial ROS Measurement

Mitochondrial ROS (mtROS) levels were measured using MitoSOX Red (M36008; Thermo Fisher Scientific, USA). The pHTMs were incubated with 5 µM MitoSOX Red for 15 minutes at 37°C, followed by imaging using a microscope (Olympus, Japan) under 63 × magnification and analysis using ImageJ software.

### Mitochondrial Membrane Potential

For the detection of mitochondrial membrane potential (Ψm), JC-1 (abs50016; Absin, China) and tetramethylrhodamine ethyl ester perchlorate (TMRE, HY-D0985A, MedChemExpress) were utilized. JC-1 probe and TMRE were prepared following the manufacturer's protocol. JC-1 probe was added to the cells and incubated at 37°C for 20 minutes. TMRE (80 nM) was added to the pHTM medium and incubated at 37°C for 15 minutes. Microscopy (Olympus, Japan) and ImageJ software were used for imaging and analysis, respectively.

### ATP Measurement

ATP production was assessed in accordance with the manufacturer's instructions (S0026; Beyotime Biotechnology, China). Briefly, the pHTMs were lysed and centrifuged at 12,000 × *g* at 4°C for 5 minutes. The resulting supernatant was added to black 96-well plates containing the ATP detection working liquid. Chemiluminescence was detected using the Nivo Multi Microplate Reader (NIVO PerkinElmer VICTOR, PerkinElmer, Finland).

### NAD^+^ Concentration Measurement

Levels of NAD^+^ in homogenates were measured following the manufacturer's instructions (S0175; Beyotime Biotechnology, China).^24^ Cells were homogenized and divided into two aliquots. One aliquot was used to measure total NAD (NADt). For NADH, samples were heated to 60°C for 30 minutes to decompose NAD^+^ following the manufacturer's instructions and previous report.^24^ A hybrid reader (NIVO PerkinElmer VICTOR Nivo Multi Microplate Reader, PerkinElmer, Finland) was used to read at optical density 450 nm at the 2-hour time point. NAD^+^ was calculated using the equation NAD^+^ = NADt − NADH.

### Transmission Electron Microscopy

For transmission electron microscopy (TEM) testing, TM tissues from mice were initially fixed with Ito's fixative, post-fixed in OsO_4_, and dehydrated according to previously described procedures.^14^ Following a 1-hour incubation with propylene oxide and resin, TM tissue was embedded in fresh resin. Ultrathin sections (50–70 nm) were cut from the tissues and incubated with saturated uranyl acetate for 15 to 30 minutes. Sections were examined using a HITACHI HT7700 120-kV TEM (Hitachi High-Tech, USA).

### IOP Measurement

After anesthesia induction with 5% isoflurane (NDC 66794-017-25; Bethlehem, PA, USA), the IOP of the mice was measured using a rebound tonometer (Tonolab Colonial Medical Supply, Londonderry, NH).^36^ IOP measurements were consistently recorded by the same researcher between 8 AM and 10 AM every week. The measured values were averaged from three repeated measurements per animal at each time point.

### Histology: Retina Whole Mount

Retina flatmount preparation has been described previously.^26^ Briefly, dissected eyeballs were post-fixed in 4% paraformaldehyde (PFA). Subsequently, retinas were blocked with 5% normal donkey serum and then incubated in RNA binding protein with multiple splicing (RBPMS, 1:1000, 15187-1-AP; Proteintech, China) overnight at 4°C and with a secondary antibody (1:1000, Alexa Fluor 488 AffiniPure Donkey Anti-Goat) for 2 hours. Retinal flatmounts were imaged with a Leica TCS SP8 X (Leica, Germany) under 20 × magnification and analyzed using ImageJ.

### Statistical Analysis

Statistical analyses were performed using Prism version 8.4.2 software (GraphPad Software Inc., La Jolla, CA, USA). Particularly, a *t*-test and 1-way analysis of variance (ANOVA) with Tukey's multiple comparison tests were conducted. Three or more biologically independent repeated experiments were completed, unless otherwise specified in this study. Normalization was used to process the data pertaining to ATP and NAD^+^ by utilizing the control group as a baseline reference for comparison. Results were considered statistically significant when *P* < 0.05, and data were presented as mean ± standard error of the mean.

## Results

### NR Attenuated DEX-Induced ECM Accumulation

The pHTMs were exposed to DEX to observe the expression of ECM-related proteins (FN, myocilin, and COL4A) and cellular function. As illustrated in Supplementary Figures S2A and S2B, Western blotting and immunofluorescence revealed a significant overexpression of ECM-related proteins, indicating abnormal ECM deposition induced by DEX. Furthermore, TM dysfunction was also a contributing factor to the pathogenesis of GIG.^37^ DEX treatment impaired the phagocytosis, migration, and cellular activity of pHTMs, consistent with the findings of other studies.^6^^,^^37^ DEX significantly inhibited phagocytosis and reduced cell proliferation compared with the control group (Supplementary Figs. S2C, S2D). These results collectively suggest that DEX induces ECM deposition and dysfunction of pHTMs in vitro.

**Figure 1.**
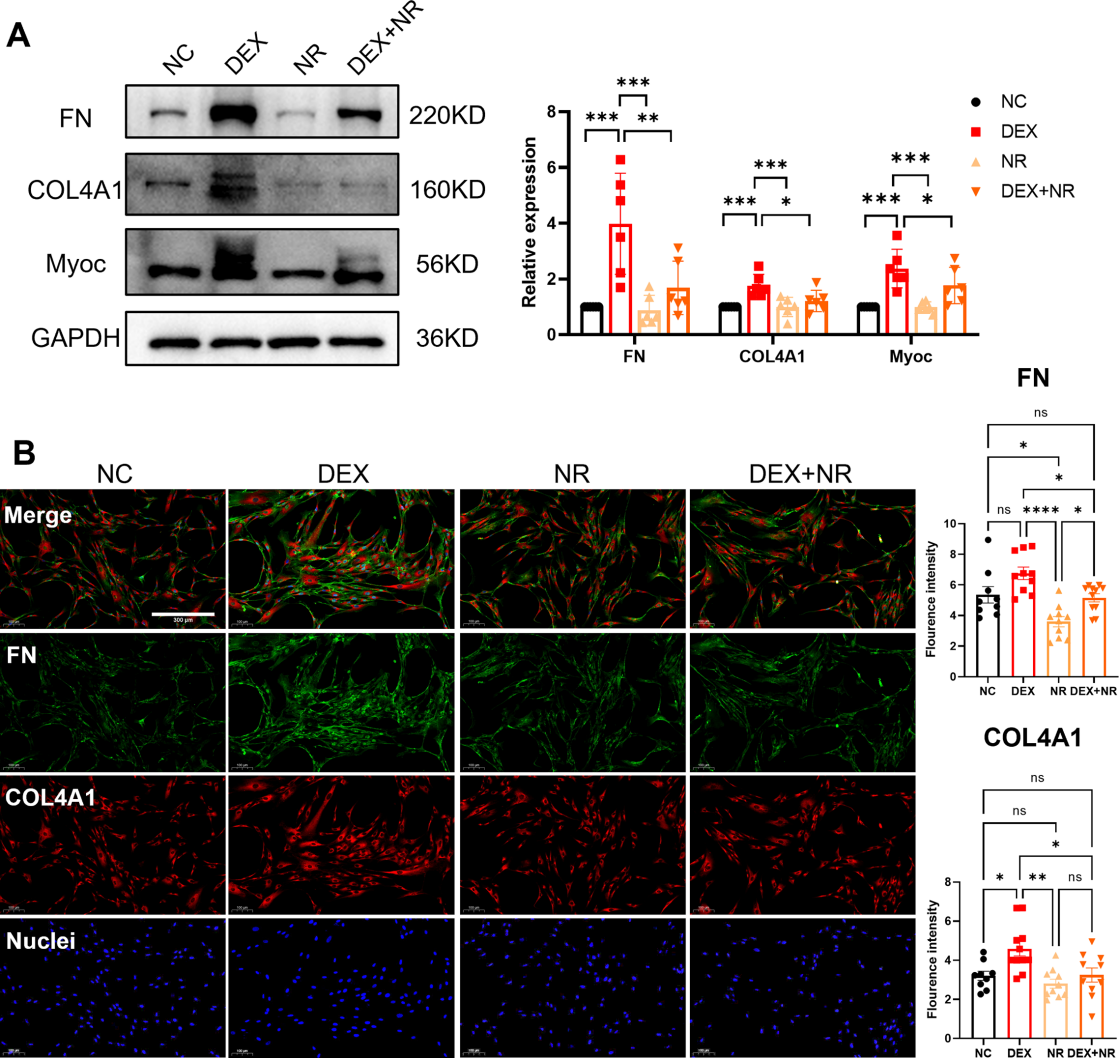
Nicotinamide riboside (NR) prevented the overexpression of the extracellular matrix (ECM) induced by DEX. (**A**) Protein levels of ECM–related markers (fibronectin [FN], COL4, and Myocilin) were assessed through Western blotting, and quantifications were performed. Data presented are from six individual Western blot experiments, which were derived from three separate donor cultures. One-way ANOVA with Tukey's multiple tests was conducted for each ECM marker, * *P* < 0.05, ** *P* < 0.01, *** *P* < 0.001, no statistical difference for unmarked comparisons. (**B**) Representative images of pHTMs stained against FN and COL4A from various treated groups, along with quantification of fluorescence intensity (*n* ≥ 9 fields per group). The results are expressed as mean ± standard error of the mean. One-way ANOVA with Tukey's multiple tests was conducted, * *P* < 0.05, ** *P* < 0.01, *** *P* < 0.001, **** *P* < 0.0001. Scale bar = 200 µm (**B**).

Figure 1 illustrates the Western blot and immunofluorescence findings, demonstrating the expression levels of ECM-related proteins in a GIG cell model treated with NR. In comparison to the DEX-only group, the NR+DEX group exhibited a significant reduction in the protein levels of FN, COL4A1, and myocilin. Furthermore, the immunofluorescence results also suggest that NR treatment prevents the overexpression of FN and COL4A1 induced by DEX. These findings suggest that NR mitigates ECM deposition induced by DEX in vitro.

### NR Prevented DEX-Induced pHTMs Dysfunction

**Figure 2.**
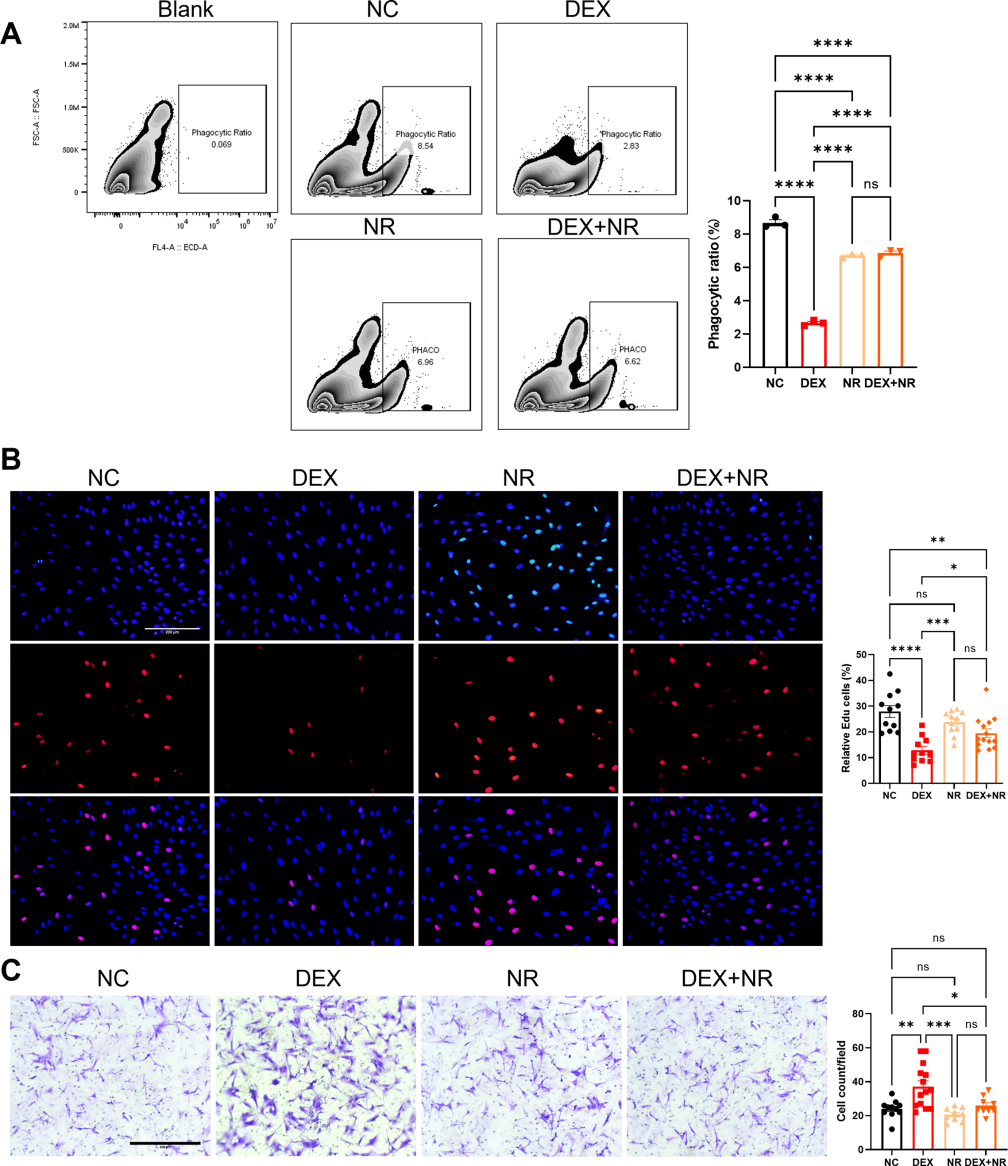
Nicotinamide riboside (NR) attenuated the dysfunction of primary human trabecular meshwork cells (pHTMs) induced by DEX in vitro. (**A**) Phagocytosis function of pHTMs was measured using flow cytometry. (**B**) Cell proliferation of pHTMs was evaluated by EdU staining. Representative images of pHTMs stained with EdU from different treated groups and quantifications of proliferative rates. (**C**) The Transwell test demonstrated that NR prevented DEX-induced increased migration of pHTMs. The results are represented as mean ± standard error of the mean. One-way ANOVA with Tukey's multiple tests, * *P* < 0.05, ** *P* < 0.01, *** *P* < 0.001, **** *P* < 0.0001. The data analyzed in Figure 3A were from 3 separate donor cultures (*n* ≥ 9 fields per groups in panels **B** and **C**). Data presented in Figures 3B and 3C represent at least three biological replicates from three separate donor cultures. Scale bar = 200 µm.

To investigate whether NR treatment could prevent DEX-induced dysfunction in pHTMs in vitro, phagocytosis, cell proliferation, and migration were assessed in a GIG cell model. Flow cytometry analyses (Fig. 2A) revealed increased intracellular beads, confirming that NR treatment enhances phagocytosis in DEX-treated pHTMs. Additionally, NR partially prevented the suppressed cellular proliferation (Fig. 2B). A previous study has indicated that the migration of pHTMs may contribute to cell loss in the aging meshwork, as well as additional cell loss in primary open-angle glaucoma.^38^ In this study, the Transwell assay was used to evaluate the migratory and invasive capabilities of pHTMs treated with DEX and NR. Although GC treatment enhanced cellular migration (Fig. 2C), NR prevented the activated migration of pHTMs induced by DEX. In summary, NR treatment not only prevents excessive ECM accumulation but also alleviates impaired pHTMs function induced by DEX.

### NR Prevented Mitochondria Morphological and Functional Changes Caused by DEX in pHTMs

**Figure 3.**
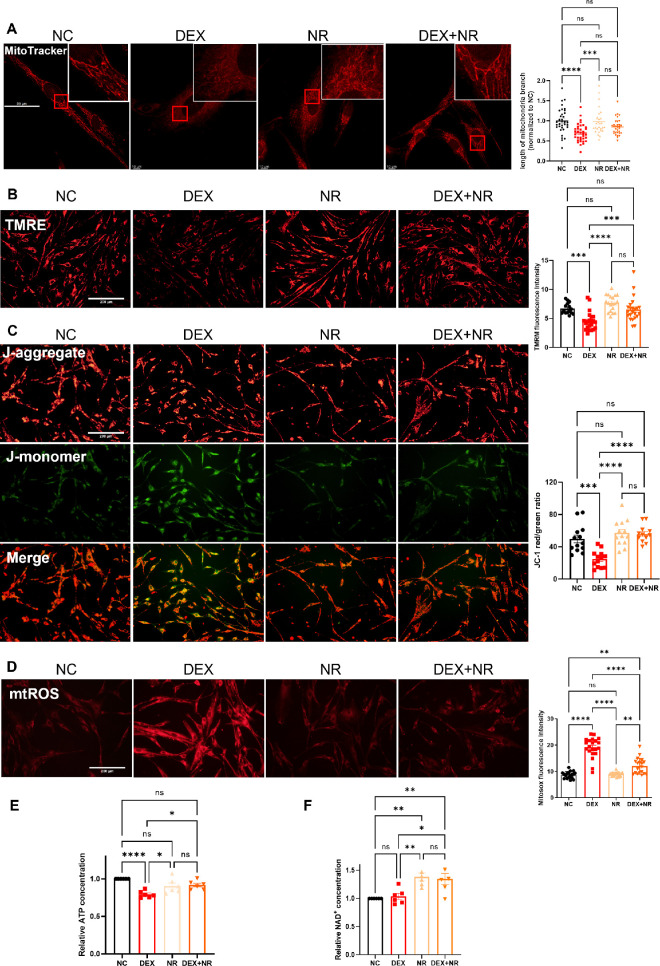
Nicotinamide riboside (NR) prevented mitochondrial damage in primary human trabecular meshwork cells (pHTMs) induced by DEX in vitro. (**A**) Mitochondrial morphology was assessed using MitoTracker dye (*n* ≥ 27 cells analyzed per groups). (**B, C**) Mitochondrial membrane potential (Ψm) was measured by tetramethylrhodamine ethyl ester perchlorate (**B**) and JC-1 staining (**C**). (**D**) Mitochondrial ROS (mtROS) was detected by MitoSOX Red Dye. (**E**) NR prevented damaged ATP production in the GIG cell model. (**F**) NR elevated intracellular NAD^+^ levels in the GIG cell model. The results are presented as mean ± standard error of the mean. One-way ANOVA with Tukey's multiple tests, * *P* < 0.05, ** *P* < 0.01, *** *P* < 0.001, **** *P* < 0.0001 (*n* ≥ 12 fields per groups in panels **B**, **C**, and **D**). Data presented in **B**, **C**, and **D** represent at least three biological replicates from three separate donor cultures. Data presented in panels **E** and **F** are two replicated samples from three independent donor cultures, totaling six samples per group. Scale bar = 50 µm (**A**), 200 µm (**B, C, D**).

The pathogenic role of mitochondrial damage in glaucoma development is supported by increasing evidence. To assess whether DEX induces mitochondrial damage, confocal microscopy and MitoTracker Red dye were utilized. Figure 3A shows that pHTMs in the control group had rod-shaped and elongated mitochondria, whereas DEX treatment might induce mitochondrial fragmentation. NR treatment partially prevented the shortened mitochondria induced by DEX, possibly protected elongated and interconnected mitochondrial networks (see Fig. 3A). Nevertheless, the incorporation of MitoTracker dyes into the mitochondrial matrix is dependent on inner membrane potential. Consequently, the reduced mitochondrial membrane potential observed in DEX-treated pHTMs may account for the shortened mitochondria. Mitochondrial fragmentation adversely affected mitochondrial function.^39^

To further investigate the effect of DEX on mitochondrial potential of pHTMs, the present study evaluated the impact of DEX on mitochondrial membrane potential using JC-1 and TMRE staining, revealing a significant decrease in Ψm after DEX treatment, reflecting by decreased J-aggregate/J-monomer (red/green) ratio and decrease in TMRE fluorescence intensity (Figs. 3B, 3C). ATP measurements, which were performed to further verify the effects of DEX on mitochondrial ATP synthesis in vitro, demonstrated impaired ATP synthesis after DEX treatment. In this study, mtROS was used to measure mitochondrial superoxide (mitosox) products in pHTMs to reflect the oxidative state of mitochondria. DEX administration led to the accumulation of mitosox in pHTMs, reflecting in the enhanced fluorescence intensity of mtROS (Fig. 3D). On the contrary, NR treatment increased Ψm (see Figs. 3B, 3C), restoring impaired mitochondrial ATP synthesis induced by DEX (Fig. 3E). Notably, NR treatment significantly prevented mitosox overproduction (see Fig. 3D). These results collectively suggest that DEX induces abnormal mitochondrial membrane potential and damaged ATP synthesis, whereas NR prevents mitochondrial damage induced by DEX.

As the primary precursor of NAD^+^, NR undergoes conversion into NAD^+^ within the cellular environment. Consequently, this study investigated whether NR confers protection to mitochondria by elevating cellular NAD^+^ levels. Analysis of the NAD^+^/NADH content revealed a significant increase in intracellular NAD^+^ levels following NR treatment, with or without DEX treatment (Fig. 3F), indicating that NR might exert its protective effects on pHTMs by elevating intracellular NAD^+^.

### NR Attenuates Glaucomatous Damage in the GIG Mouse Model

**Figure 4.**
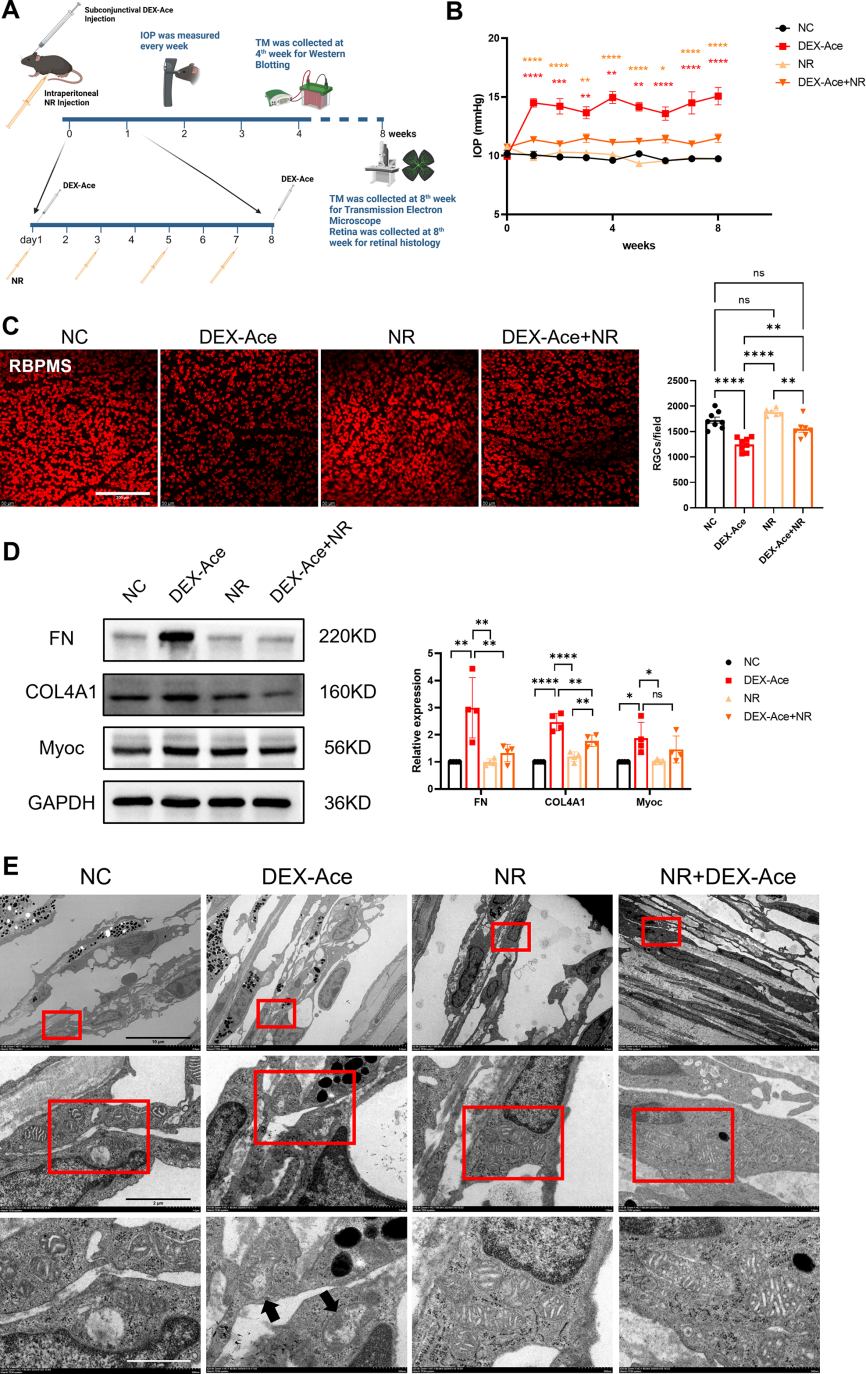
Nicotinamide riboside (NR) prevented the progression of GIG in vivo. (**A**) Schematic course of GIG mouse model building and NR treatment. (**B**) NR partially prevented increased intraocular pressure in the GIG mouse model (*red asterisk =* NC versus DEX-Ace, and the *orange asterisk =* DEX-Ace versus DEX-Ace+NR; *n* ≥ 12 eyes per groups). One-way ANOVA with Tukey's multiple tests was conducted for each time point, * *P* < 0.05, ** *P* < 0.01, *** *P* < 0.001, **** *P* < 0.0001. (**C**) NR protected against RGC loss in the GIG cell model. Representative images of retina flat-mounts stained against RBPMS from different treated groups. Images were randomly chosen from the region with a distance to the optic nerve of 1.0 mm (*n* = 6–8 retinas per group). One-way ANOVA with Tukey's multiple tests, ** *P* < 0.01, **** *P* < 0.0001. (**D**) NR depressed the overexpression of ECM-related proteins (FN and COL4A), as tested by Western blotting (*n* = 4 mice per group). One-way ANOVA with Tukey's multiple tests was conducted for each ECM marker, * *P* < 0.05, ** *P* < 0.01, *** *P* < 0.001, no statistical difference for unmarked comparisons. (**E**) Mitochondrias in the TM region were imaged by electron microscopy. *Black arrows* indicate damaged mitochondria in the DEX-Ace treated group. The results are expressed as mean ± standard error of the mean. Scale bar = 100 µm (**C**), 10 µm (*upper* **E**), 1 µm (*middle* **E**), and 500 nm (*lower* **E**).

Based on in vitro data, this study confirms that NR could notably prevent ECM accumulation as well as dysfunction of pHTMs, protecting against mitochondrial damage in the GIG cell model. To further investigate the protection of NR, this study established a GIG mouse model by subconjunctival injection of DEX-Ace to mimic the onset of GIG (Fig. 4A). As shown in Figure 4B, IOP increased immediately after the first administration of DEX-Ace in the GIG group and stayed elevated after continuous injection of DEX-Ace compared with the baseline. As shown in Figure 4C, RBPMS staining revealed that continuous DEX-Ace injection for 8 weeks resulted in a significant loss of RGCs. Furthermore, IOP elevation after DEX-Ace treatment is believed to result from ECM deposition, which was tested by Western blot to clarify ECM changes in the TM. Western blot results showed that ECM-related proteins (FN and COL4A) in DEX-treated mice were overexpressed compared with the control group (Fig. 4D). Compared with the GIG-only group, mice in the NR-treated group showed noticeable improvement in glaucomatous changes, including IOP elevation, ECM deposition, and RGC loss (see Figs. 4B–D). In summary, NR treatment could prevent glaucomatous damage in the GIG mouse model.

This study then assessed the essential role of mitochondria in GIG and NR protection. TEM was performed to identify mitochondrial morphological changes 8 weeks after DEX-Ace treatment in the mouse TM region. Compared with the control group, the ultrastructure of TM in the DEX-Ace-treated group showed increased mitochondrial fragmentation and damage. By contrast, NR treatment could partially prevent the mitochondrial ultrastructure damage in vivo (Fig. 4E).

## Discussion

Mitochondrial damage occurs in TM following DEX treatment.^6^ Accumulating evidence suggests the importance of mitochondria in the pathogenesis of GCs.^6^^,^^7^ Our current study reveals that DEX induces abnormal mitochondrial fragmentation, damaged ATP synthesis, decreased Ψm, and overexpression of mitochondrial ROS in TM. Meanwhile, NR mitigates DEX-induced damage, including ECM accumulation and dysfunction in pHTMs, by preventing mitochondrial membrane potential and protect against overproduction of mitochondrial superoxide. In addition, NR prevented damaged ATP synthesis and overexpression of mitochondrial ROS, improving Ψm in the GIG cell model. Unexpectedly, our flow cytometry analysis of phagocytosis experiments revealed a significant decrease in the phagocytic ability of cells treated solely with NR compared to the control group (see Fig. 2A). We speculate that DMSO, which was used in the preparation of the NR storage solution, may have an impact on the phagocytic function of the cells, or that NR (400 µm) may affect cell phagocytosis. Further research is needed to conduct additional tests to validate our hypotheses. Additionally, elevated intracellular NAD^+^ levels after NR administration were also detected, suggesting that NR might exert protection by increasing cellular NAD^+^ levels.

GCs exert their main action through GR, which are nuclear receptors activated by ligands, resulting in conformational changes in GR.^40^^–^^42^ Ultimately, this reshapes gene expression patterns and leads to changes in the microstructure and function of TM cells, including inhibition of phagocytosis, alterations in cellular junctional complexes, and abnormal ECM deposition.^43^^,^^44^ All these changes alter TM stiffness, which correlates positively with outflow resistance.^4^^,^^45^ The mechanical properties of TM are closely related to the function of the outflow pathway, ultimately influencing IOP.^45^ Additional studies have demonstrated that Schlemm's canal endothelial cells (SCECs) affected by glaucoma exhibit heightened ECM levels, increased proliferation and migration rates, and decreased mitochondrial activity compared to healthy SCECs.^46^ It is suggested that NR may potentially mitigate IOP elevation by mitigating abnormal ECM expression or enhancing mitochondrial function in SCECs within the conventional outflow pathway, thereby influencing distal outflow pathway regulation. Nevertheless, the validity of this hypothesis necessitates confirmation through forthcoming investigations. This study confirmed excessive ECM deposition and elevated IOP in the TM region in the GIG mouse model (see Fig. 4). By contrast, NR treatment partially prevents ECM accumulation and GIG progression.

In addition to abnormal ECM accumulation, GC-induced TM dysfunction is also believed to contribute to GIG.^47^ Impaired phagocytosis and reduced proliferation were detected in pHTMs after DEX treatment in this study, whereas NR notably prevents pHTMs dysfunction (see Fig. 2). This indicates that NR protects against glaucomatous damage by reducing ECM deposition and enhancing TM function. However, the exact mechanism of increased ECM deposition and dysfunction after GC treatment has not been elucidated thus far.

Mitochondria play a crucial role in ECM remodeling and the pathogenesis of GIG.^6^^,^^11^ The presence of GCs causes mitochondrial damage, including a halt in motility, excessive mitochondrial fission through Drp1 phosphorylation, impaired Ca^2+^ buffering, and excessive production of mtROS.^48^ Mitochondrial damage leads to increased ROS generation.^49^ Our study further highlights that mitochondrial damage, such as decrease in Ψm and damaged ATP synthesis, is pivotal in the progression of GIG. DEX induces reduced Ψm and impaired ATP production, resulting in the overproduction of mtROS. Mitochondria have been reported to be closely related to both ECM deposition and degradation.^10^ When mtROS is released into the cytosol, it activates various pro-inflammatory mediators, subsequently altering the expression and regulation of matrix metalloproteinases (MMPs) and tissue inhibitors of MMPs, thereby causing changes in the ECM.^50^ DEX downregulates the expression of MMP-3 and MMP-13 while upregulating the expression of Link protein and FN.^51^ Apart from ECM deposition, the oxidation-reduction state, controlled by the rate of mitochondrial oxidant production, can also affect cell differentiation and proliferation.^52^ This study demonstrated that NR could prevent decrease in Ψm and protect against mtROS overproduction. Based on these results, NR may exert protective effects on ECM accumulation and dysfunction of TM by attenuating mitochondrial ATP production, impaired Ψm, and ROS production, thereby remodeling the ECM.

As clinical trials concerning NR advance, its therapeutic potential is increasingly being elucidated in a wider scope.^53^^,^^54^ In existing studies, NR has demonstrated potential therapeutic effects on various diseases, with a particular focus on metabolic, neurodegenerative, and age-related conditions.^22^^,^^55^ Furthermore, in both animal models and clinical trials related to glaucoma, NAD^+^ has exhibited protective effects on RGCs^24^ and preserved visual function in patients with glaucoma.^27^ Based on the effects of NR on the ECM of TM cells and its IOP-lowering effect observed in this study, along with its potential influence on other outflow pathways, it is hypothesized that NR may prevent the rise in IOP by modulating the ECM of TM. In summary, NR holds promise as a novel therapeutic strategy for GIG.

However, in this study, the effects of NR on DEX induced ECM and mitochondria of TM were observed only when NR was administrated in conjunction with DEX. Future research should focus on validating the protective properties of NR at different treatment intervals, thus advancing our comprehension of NR's potential for clinical application.

## Conclusions

This study aimed to evaluate the protective effects of NR on TM in GIG using both in vitro and in vivo approaches. Our findings revealed that NR effectively prevented the overexpression of ECM-related proteins, restoring TM function and impeding the progression of GIG. The potential mechanism behind NR's protection may include the mitigation of mitochondrial damage. However, it is crucial to acknowledge that NR's protective mechanisms in ECM accumulation may involve multiple pathways concurrently. Thus, additional evidence is necessary for a comprehensive understanding of the pathogenesis and underlying mechanisms of NR's protection in TM. In conclusion, our study suggests that NR holds promising potential as a therapeutic candidate for treating TM damage in GIG.

## Supplementary Material

Supplement 1

## References

[1] Vandewalle J, Luypaert A, De Bosscher K, Libert C. Therapeutic mechanisms of glucocorticoids. *Trends Endocrinol Metab*. 2018; 29(1): 42–54. 29162310 10.1016/j.tem.2017.10.010

[2] Kato M, Hosoe N, Gotoda T, et al. Treatment with vonoprazan for 3 weeks is not inferior to 8 weeks for the management of gastric ESD: a multicenter noninferiority randomized study. *J Gastroenterol*. 2023; 58(4): 358–366. 36781490 10.1007/s00535-023-01966-z

[3] Roy Chowdhury U, Hann CR, Stamer WD, Fautsch MP. Aqueous humor outflow: dynamics and disease. *Invest Ophthalmol Vis Sci*. 2015; 56(5): 2993–3003. 26024085 10.1167/iovs.15-16744 PMC4432549

[4] Fini ME, Schwartz SG, Gao X, et al. Steroid-induced ocular hypertension/glaucoma: focus on pharmacogenomics and implications for precision medicine. *Prog Retin Eye Res*. 2017; 56: 58–83. 27666015 10.1016/j.preteyeres.2016.09.003 PMC5237612

[5] Wang K, Read AT, Sulchek T, Ethier CR. Trabecular meshwork stiffness in glaucoma. *Exp Eye Res*. 2017; 158: 3–12. 27448987 10.1016/j.exer.2016.07.011 PMC6501821

[6] Zeng W, Wang W, Wu S, et al. Mitochondria and autophagy dysfunction in glucocorticoid-induced ocular hypertension/glaucoma mice model. *Curr Eye Res*. 2020; 45(2): 190–198. 31425668 10.1080/02713683.2019.1657462

[7] He JN, Zhang SD, Qu Y, et al. Rapamycin removes damaged mitochondria and protects human trabecular meshwork (TM-1) cells from chronic oxidative stress. *Mol Neurobiol*. 2019; 56(9): 6586–6593. 30903531 10.1007/s12035-019-1559-5

[8] Casagrande S, Stier A, Monaghan P, et al. Increased glucocorticoid concentrations in early life cause mitochondrial inefficiency and short telomeres. *J Exp Biol*. 2020; 223(Pt 15): jeb222513. 32532864 10.1242/jeb.222513

[9] Lee SR, Kim HK, Song IS, et al. Glucocorticoids and their receptors: insights into specific roles in mitochondria. *Prog Biophys Mol Biol*. 2013; 112(1-2): 44–54. 23603102 10.1016/j.pbiomolbio.2013.04.001

[10] Cai L, Shi L, Peng Z, Sun Y, Chen J. Ageing of skeletal muscle extracellular matrix and mitochondria: finding a potential link. *Ann Med*. 2023; 55(2): 2240707. 37643318 10.1080/07853890.2023.2240707 PMC10732198

[11] Bubb K, Holzer T, Nolte JL, et al. Mitochondrial respiratory chain function promotes extracellular matrix integrity in cartilage. *J Biol Chem*. 2021; 297(4): 101224. 34560099 10.1016/j.jbc.2021.101224 PMC8503590

[12] Bueno M, Calyeca J, Rojas M, Mora AL. Mitochondria dysfunction and metabolic reprogramming as drivers of idiopathic pulmonary fibrosis. *Redox Biol*. 2020; 33: 101509. 32234292 10.1016/j.redox.2020.101509 PMC7251240

[13] Siekacz K, Piotrowski WJ, Iwański MA, Górski P, Białas AJ. The role of interaction between mitochondria and the extracellular matrix in the development of idiopathic pulmonary fibrosis. *Oxid Med Cell Longev*. 2021; 2021: 9932442. 34707784 10.1155/2021/9932442 PMC8545566

[14] Zhu X, Wu S, Zeng W, et al. Protective effects of rapamycin on trabecular meshwork cells in glucocorticoid-induced glaucoma mice. *Front Pharmacol*. 2020; 11: 1006. 32714192 10.3389/fphar.2020.01006 PMC7344368

[15] Yoshino J, Baur JA, Imai SI. NAD(+) intermediates: the biology and therapeutic potential of NMN and NR. *Cell Metab*. 2018; 27(3): 513–528. 29249689 10.1016/j.cmet.2017.11.002 PMC5842119

[16] Chini CCS, Zeidler JD, Kashyap S, Warner G, Chini EN. Evolving concepts in NAD(+) metabolism. *Cell Metab*. 2021; 33(6): 1076–1087. 33930322 10.1016/j.cmet.2021.04.003 PMC8172449

[17] Goody MF, Henry CA. A need for NAD+ in muscle development, homeostasis, and aging. *Skelet Muscle*. 2018; 8(1): 9. 29514713 10.1186/s13395-018-0154-1 PMC5840929

[18] Guo J, Zhou Y, Xu C, et al. Genetic determinants of somatic selection of mutational processes in 3,566 human cancers. *Cancer Res*. 2021; 81(16): 4205–4217. 34215622 10.1158/0008-5472.CAN-21-0086 PMC9662923

[19] Romani M, Sorrentino V, Oh CM, et al. NAD(+) boosting reduces age-associated amyloidosis and restores mitochondrial homeostasis in muscle. *Cell Rep*. 2021; 34(3): 108660. 33472069 10.1016/j.celrep.2020.108660 PMC7816122

[20] Wang S, Wan T, Ye M, et al. Nicotinamide riboside attenuates alcohol induced liver injuries via activation of SirT1/PGC-1alpha/mitochondrial biosynthesis pathway. *Redox Biol*. 2018; 17: 89–98. 29679894 10.1016/j.redox.2018.04.006 PMC6007172

[21] Zhou B, Ding-Hwa Wang D, Qiu Y, et al. Boosting NAD level suppresses inflammatory activation of PBMCs in heart failure. *J Clin Invest*. 2020; 130(11): 6054–6063. 32790648 10.1172/JCI138538 PMC7598081

[22] Pham TX, Bae M, Kim MB, et al. Nicotinamide riboside, an NAD+ precursor, attenuates the development of liver fibrosis in a diet-induced mouse model of liver fibrosis. *Biochim Biophys Acta Mol Basis Dis*. 2019; 1865(9): 2451–2463. 31195117 10.1016/j.bbadis.2019.06.009 PMC6614025

[23] Faivre A, Katsyuba E, Verissimo T, et al. Differential role of nicotinamide adenine dinucleotide deficiency in acute and chronic kidney disease. *Nephrol Dial Transplant*. 2021; 36(1): 60–68. 33099633 10.1093/ndt/gfaa124

[24] Zhang X, Zhang N, Chrenek MA, et al. Systemic treatment with nicotinamide riboside is protective in two mouse models of retinal ganglion cell damage. *Pharmaceutics*. 2021; 13(6): 893. 34208613 10.3390/pharmaceutics13060893 PMC8235058

[25] Li Y, Zhang N, Zhang X, et al. Oral supplementary nicotinamide riboside treats several components of the DBA/2J murine model of pigment dispersion glaucoma. Presented at the ARVO Annual Meeting Abstract, June 2022. *Invest Ophthalmol Vis Sci*. 2022; 63(7): 2375-A0059–2375-A0059.

[26] Zhang N, Li Y, Zhang X, et al. Systemic nicotinamide riboside treatment protects retinal ganglion cells in an aged optic nerve crush mouse model. Presented at the ARVO Annual Meeting Abstract, June 2022. *Invest Ophthalmol Vis Sci*. 2022; 63(7): 2717-A0081–2717-A0081.

[27] Hui F, Tang J, Williams PA, et al. Improvement in inner retinal function in glaucoma with nicotinamide (vitamin B3) supplementation: a crossover randomized clinical trial. *Clin Exp Ophthalmol*. 2020; 48(7): 903–914. 32721104 10.1111/ceo.13818

[28] Gustavsson ST, Enz TJ, Tribble JR, et al. Nicotinamide prevents retinal vascular dropout in a rat model of ocular hypertension and supports ocular blood supply in glaucoma patients. *Invest Ophthalmol Vis Sci*. 2023; 64(14): 34. 10.1167/iovs.64.14.34 PMC10683769 38010699

[29] Leung CKS, Ren ST, Chan PPM, et al. Nicotinamide riboside as a neuroprotective therapy for glaucoma: study protocol for a randomized, double-blind, placebo-control trial. *Trials*. 2022; 23(1): 45. 35039056 10.1186/s13063-021-05968-1 PMC8762963

[30] Masihzadeh O, Ammar DA, Lei TC, Gibson EA, Yahook MY. Real-time measurements of nicotinamide adenine dinucleotide in live human trabecular meshwork cells: effects of acute oxidative stress. *Exp Eye Res*. 2011; 93(3): 316–320. 21354135 10.1016/j.exer.2011.02.012 PMC5042686

[31] Patel GC, Phan TN, Maddineni P, et al. Dexamethasone-induced ocular hypertension in mice: effects of myocilin and route of administration. *Am J Pathol*. 2017; 187(4): 713–723. 28167045 10.1016/j.ajpath.2016.12.003 PMC5397678

[32] Fan Y, Wei J, Guo L, et al. Osthole reduces mouse IOP associated with ameliorating extracellular matrix expression of trabecular meshwork cell. *Invest Ophthalmol Vis Sci*. 2020; 61(10): 38. 10.1167/iovs.61.10.38 PMC7445364 32821914

[33] Keller KE, Bhattacharya SK, Borrás T, et al. Consensus recommendations for trabecular meshwork cell isolation, characterization and culture. *Exp Eye Res*. 2018; 171: 164–173. 29526795 10.1016/j.exer.2018.03.001 PMC6042513

[34] Clark AF, Steely HT, Dickerson JE Jr, et al. Glucocorticoid induction of the glaucoma gene MYOC in human and monkey trabecular meshwork cells and tissues. *Invest Ophthalmol Vis Sci*. 2001; 42(8): 1769–1780. 11431441

[35] Valente AJ, Maddalena LA, Robb EL, Moradi F, Stuart JA. A simple ImageJ macro tool for analyzing mitochondrial network morphology in mammalian cell culture. *Acta Histochem*. 2017; 119(3): 315–326. 28314612 10.1016/j.acthis.2017.03.001

[36] Struebing FL, King R, Li Y, et al. Genomic loci modulating retinal ganglion cell death following elevated IOP in the mouse. *Exp Eye Res*. 2018; 169: 61–67. 29421330 10.1016/j.exer.2017.12.013 PMC5939594

[37] Yoo H, Singh A, Li H, et al. Simvastatin attenuates glucocorticoid-induced human trabecular meshwork cell dysfunction via YAP/TAZ inactivation. *Curr Eye Res*. 2023; 48(8): 736–749. 37083467 10.1080/02713683.2023.2206067 PMC10524554

[38] Hogg P, Calthorpe M, Batterbury M, Grierson I. Aqueous humor stimulates the migration of human trabecular meshwork cells in vitro. *Invest Ophthalmol Vis Sci*. 2000; 41(5): 1091–1098. 10752946

[39] Meyer JN, Leuthner TC, Luz AL. Mitochondrial fusion, fission, and mitochondrial toxicity. *Toxicology*. 2017; 391: 42–53. 28789970 10.1016/j.tox.2017.07.019 PMC5681418

[40] Patel GC, Millar JC, Clark AF. Glucocorticoid receptor transactivation is required for glucocorticoid-induced ocular hypertension and glaucoma. *Invest Ophthalmol Vis Sci*. 2019; 60(6): 1967–1978. 31050723 10.1167/iovs.18-26383 PMC6890434

[41] Patel PD, Kodati B, Clark AF. Role of glucocorticoids and glucocorticoid receptors in glaucoma pathogenesis. *Cells*. 2023; 12(20): 2452. 37887296 10.3390/cells12202452 PMC10605158

[42] Kokkinopoulou I, Moutsatsou P. Mitochondrial glucocorticoid receptors and their actions. *Int J Mol Sci*. 2021; 22(11): 6054. 34205227 10.3390/ijms22116054 PMC8200016

[43] Zhang X, Ognibene CM, Clark AF, Yorio T. Dexamethasone inhibition of trabecular meshwork cell phagocytosis and its modulation by glucocorticoid receptor beta. *Exp Eye Res*. 2007; 84(2): 275–284. 17126833 10.1016/j.exer.2006.09.022 PMC1796996

[44] Stamer WD, Clark AF. The many faces of the trabecular meshwork cell. *Exp Eye Res*. 2017; 158: 112–123. 27443500 10.1016/j.exer.2016.07.009 PMC5247412

[45] Wang K, Li G, Read AT, et al. The relationship between outflow resistance and trabecular meshwork stiffness in mice. *Sci Rep*. 2018; 8(1): 5848. 29643342 10.1038/s41598-018-24165-w PMC5895808

[46] Kelly RA, Perkumas KM, Campbell M, et al. Fibrotic changes to Schlemm's canal endothelial cells in glaucoma. *Int J Mol Sci*. 2021; 22(17):9446. 10.3390/ijms22179446 PMC8431431 34502356

[47] Faralli JA, Desikan H, Peotter J, et al. Genomic/proteomic analyses of dexamethasone-treated human trabecular meshwork cells reveal a role for GULP1 and ABR in phagocytosis. *Mol Vis*. 2019; 25: 237–254. 31516309 PMC6706170

[48] Tome ME, Lee K, Jaramillo MC, Briehl MM. Mitochondria are the primary source of the H(2)O(2) signal for glucocorticoid-induced apoptosis of lymphoma cells. *Exp Ther Med*. 2012; 4(2): 237–242. 22844350 10.3892/etm.2012.595 PMC3404723

[49] Annesley SJ, Fisher PR. Mitochondria in health and disease. *Cells*. 2019; 8(7): 680. 31284394 10.3390/cells8070680 PMC6678092

[50] Piantadosi CA, Suliman HB. Mitochondrial dysfunction in lung pathogenesis. *Annu Rev Physiol*. 2017; 79: 495–515. 27959621 10.1146/annurev-physiol-022516-034322

[51] Richardson DW, Dodge GR. Dose-dependent effects of corticosteroids on the expression of matrix-related genes in normal and cytokine-treated articular chondrocytes. *Inflamm Res*. 2003; 52(1): 39–49. 12608648 10.1007/s000110300012

[52] Weinberg SE, Sena LA, Chandel NS. Mitochondria in the regulation of innate and adaptive immunity. *Immunity*. 2015; 42(3): 406–417. 25786173 10.1016/j.immuni.2015.02.002 PMC4365295

[53] Helman T, Braidy N. Importance of NAD+ anabolism in metabolic, cardiovascular and neurodegenerative disorders. *Drugs Aging*. 2023; 40(1): 33–48. 36510042 10.1007/s40266-022-00989-0

[54] Sharma C, Donu D, Cen Y. Emerging role of nicotinamide riboside in health and diseases. *Nutrients*. 2022; 14(19): 3889. 36235542 10.3390/nu14193889 PMC9571518

[55] Iqbal T, Nakagawa T. The therapeutic perspective of NAD(+) precursors in age-related diseases. *Biochem Biophys Res Commun*. 2024; 702: 149590. 38340651 10.1016/j.bbrc.2024.149590

