# Supplementary material for: Protective Effect of Nicotinamide Riboside on Glucocorticoid-Induced Glaucoma: Mitigating Mitochondrial Damage and Extracellular Matrix Deposition
Source: Invest Ophthalmol Vis Sci. 2024 Jul 1;65(8):1. doi: 10.1167/iovs.65.8.1 (PMC11221610; doi:10.1167/iovs.65.8.1)
Supplement: Supplement 1 [file iovs-65-8-1_s001.pdf]

Supplementary material

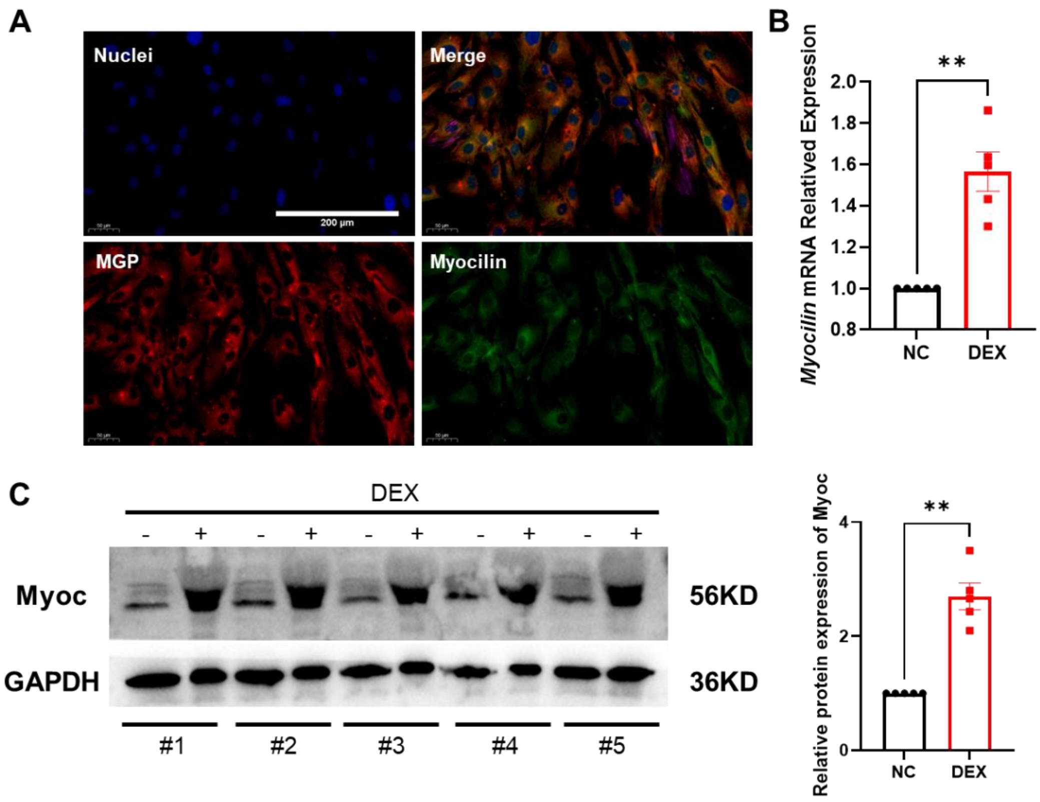

*Supplementary Figure 1. Cell identification of primary human trabecular meshwork cells. A. Myocilin and Matrix Gla Protein (MGP) were used for cell identification. Scale bar: 200 µm. B. mRNA expression of myocilin for 5 donor cultures response to Dexamethasone. B. Protein expression of myocilin for 5 donor cultures response to Dexamethasone. n=5. Paired t-test, ** P<0.01. Mean ± Standard error of mean.*

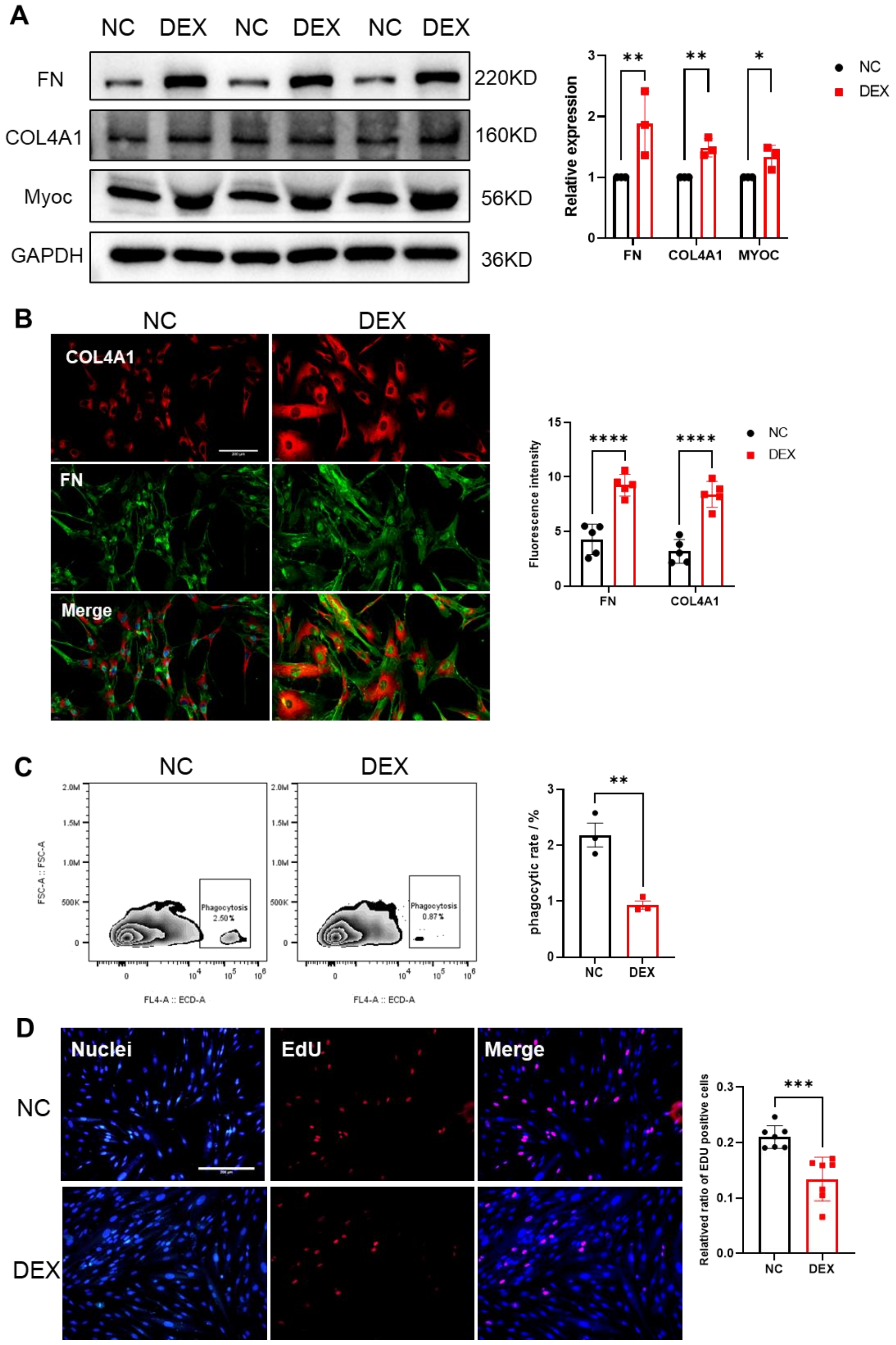

Supplementary Figure 2. Dexamethasone (DEX) induced extracellular matrix (ECM) deposition and dysfunction of primary human trabecular meshwork cells (pHTMs) ex vivo. (A) Protein levels of ECM-related markers (fibronectin [FN], collagen [COL]4A, and

*myocilin) were assessed by western blot and quantified, the 3 groups of samples analyzed in 1A were from 3 separate donor cultures. (B) Representative images of pHTMs stained against FN and COL4A from the control and DEX-treated groups. 5 fields per group. (C) The phagocytotic assay revealed depressed phagocytosis of pHTMs after DEX treatment, the 3 groups of samples analyzed in 1A were from 3 separate donor cultures. (D) Representative images of cell proliferation of pHTMs were assessed by EdU staining, demonstrating decreased cell activity induced by DEX. 7 fields per group. Data presented in this figure represent three biological replicates. The results are presented as mean ± standard error of the mean. One-way ANOVA with Tukey's multiple test, ** p<0.01, *** p<0.001, **** p<0.001. Scale bar: 200 μm.*

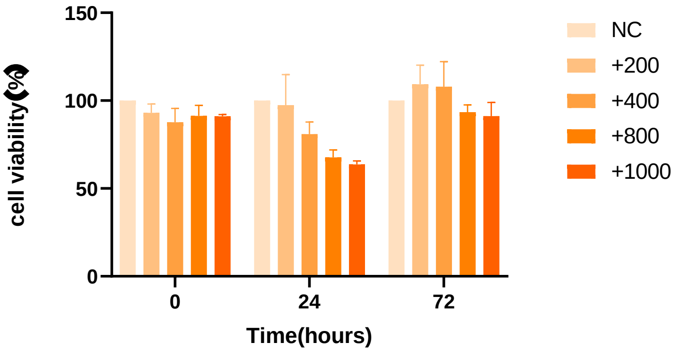

Supplementary Figure 3. The effect of different concentrations of NR on primary human trabecular meshwork cells' viability after 24 and 72 hours of treatment, was determined by using the CCK-8 assay. The results are expressed as mean ± standard error of the mean. One-way analysis of variance with Tukey's multiple tests. No significant differences were observed between each group at each time point.
